# What is a ‘serious’ genetic condition? The perceptions of people living with genetic conditions

**DOI:** 10.1038/s41431-021-00962-2

**Published:** 2021-09-27

**Authors:** Felicity K. Boardman, Corinna C. Clark

**Affiliations:** Warwick Medical School, University of Warwick, CV4 7HL Coventry, UK

**Keywords:** Genetic counselling, Ethics, Quality of life

## Abstract

Despite no consensus on the definition of ‘seriousness’, the concept is regularly used in policy and practice contexts to categorise conditions, determine access to genetic technologies and uses of selective pregnancy termination. Whilst attempts have been made to create taxonomies of genetic condition seriousness to inform clinical and policy decision-making, these have often relied on condition appraisals made by health and genetics professionals. The views of people with genetic conditions have been largely under-represented. This study explores the concept of seriousness through the perspectives of people with a range of ‘clinically serious’ conditions (fragile X conditions, spinal muscular atrophy, cystic fibrosis, haemophilia, thalassaemia). Attitudes towards suffering, quality of life (QoL) and selective pregnancy termination were elucidated from 45 in-depth qualitative interviews and 469 postal/online surveys. The majority of participants reported good health/wellbeing, and the capacity for good QoL, despite experiencing suffering with their condition. Notably, participants with later-onset conditions held more negative views of their health and QoL, and were more likely to view their condition as an illness, than those with early-onset conditions. These participants were more likely to see their condition as part of their identity. Whilst most participants supported prenatal screening, there was little support for selective termination. Moreover, social environment emerged as a critical mediator of the experience of the condition. The complex and rich insights of people living with genetic conditions might usefully be incorporated into future genetic taxonomies of ‘seriousness’ to ensure they more accurately reflect the lived reality of those with genetic conditions.

## Introduction

As an era of targeted genetic testing gives way to global mainstreaming of high output genomic sequencing, important decisions need to be made regarding which genetic variants, associated with which conditions, are considered ‘worth knowing’ [1]. In addition to the strength of genotype/phenotype correlations, the availability of effective treatment/prevention and significance of the genetic findings to wider family members [2], the ‘seriousness’ of conditions has been pivotal to policy decisions governing the uses of genetic screening [3]. The notion of ‘seriousness’ has been employed to guide professionals [4, 5] and patients [6] in determining which incidental findings should be returned from whole genome sequencing and which variants should be included on population level genetic screening panels [3, 7]. It is also employed to govern access to selective pregnancy termination and technologies such as pre-implantation genetic diagnosis (PGD). However, what ‘counts’ as a serious condition remains contested, with no clear legal or social definition [8, 9].

Building on guidelines produced by the American College of Medical Genetics [10], various attempts have been made to operationalise the concept of ‘seriousness’ by researchers [6, 11–13], authoritative bodies [14], and working groups [15] in order to support clinical and policy decision-making. These attempts often draw on the views of professionals (e.g. obstetricians, genetics professionals, paediatricians), to delineate core ‘dimensions’ of seriousness (e.g. age of onset, lifespan, variability of symptoms, effective treatment). Dimension thresholds are then assigned to higher order categories (e.g. ‘mild’, ‘moderate’, ‘severe’, or ‘profound’) [6], onto which genetic conditions are then mapped. The resulting taxonomies [5, 6] are attractive in their ability to syphon complex conditions into discrete manageable categories and transform unfamiliar rare disease names into accessible lay language. Designed to streamline reproductive genomic decision-making, they enable patients to decide whether to receive genetic results for groups of similarly serious conditions, with the ultimate goal of facilitating informed decision-making [6], and to guide decisions regarding the inclusion of conditions into expanded carrier screening programmes. However, concerns remain regarding their applicability to unpredictable, fluctuating, and spectrum conditions [5], as well as their neglect of non-clinical factors (e.g. social/environmental contexts) [9, 16]. Moreover, genetics professionals indicate a preference for genetics patients to co-produce definitions of seriousness [8].

The importance of including the perspectives of people living with genetic conditions in understanding the subjective nature of ‘seriousness’ is increasingly acknowledged [7, 17–21]. Indeed, there are examples in the literature of people with disabilities evaluating their condition and quality of life (QoL) differently—and often more positively—than others around them do, suggesting differences in condition appraisal between those with and without lived experience [2, 22–24]. Disability focused research, however, has been slow to infiltrate the literature on reproductive genetics, despite the fact that people living with the conditions that screening programmes could potentially identify, arguably have the most accurate and intimate understanding of life with a genetic condition [20, 25].

This paper examines the perspectives and experiences of adults living with highly contrasting genetic conditions—including conditions that are treatable (haemophilia, thalassaemia), those which are life-limiting (cystic fibrosis, spinal muscular atrophy), those involving cognitive (fragile X syndrome) and/or physical impairment (spinal muscular atrophy, thalassaemia) (Table 1). Of these, Thalassaemia is the only condition routinely screened for prenatally in the UK. Perceptions of the ‘seriousness’ are established through an analysis of 45 in-depth interviews and 469 surveys with adults diagnosed with these conditions, that explore their perceptions of suffering, QoL, health, and attitudes towards prenatal screening (PNS) (and selective termination) for their condition. Mixed methods techniques are employed to combine the two datasets. The use of mixed methods allows a detailed exploration of participant attitudes, whilst also including a large number of participants, which is particularly beneficial when analysing such complex and nuanced topics. Finally, participant reflections on condition seriousness are positioned in the context of the broader literature and existing taxonomies [26], in order to highlight their relevance and necessary contribution to future applications of reproductive genomic medicine.

**Table 1:** Summary of conditions.

Condition Name	Description
Spinal Muscular Atrophy (SMA)	Neuromuscular disorder. SMA1 is generally fatal in infancy. SMA2 (onset 6–18 months) typified by ability to sit but not walk, many attain a near-normal lifespan. SMA3 and SMA4 (childhood and adult onset, respectively), typified by progressive muscle weakness, lifespan unaffected.
Fragile X Syndrome (FXS)	Most common cause of inherited intellectual disability. Severity and combination of symptoms varies widely (e.g. can include ADHD and epilepsy). Males are more severely affected (X-linked)
Fragile X‐associated Primary Ovarian Insufficiency (FXPOI) and Fragile X‐associated Tremor/Ataxia (FXTAS)	Affects some FXS ‘premutation’ carriers: FXPOI (women), causing reduced fertility and early menopause; FXTAS (men and women), a late-onset (>50 years) progressive neurodegenerative disorder.
Thalassaemia	Affects haemoglobin production. Only condition for which universal PNS is available in the UK. Individuals with lifetime access to blood transfusions/ and chelation therapy can achieve a near-normal lifespan. Co-morbidities include heart, bone, and endocrine problems, diabetes, cirrhosis, gallstones and reduced fertility.
Cystic Fibrosis (CF)	Most common inherited life-limiting disease in European populations (UK life expectancy 47 years). Newborn screening offered (UK) since 2007, with cascade carrier screening and PNS for affected families. Thickened epithelial secretions cause progressive damage to the lungs, intestine, liver, kidneys, pancreas, and reproductive organs. Respiratory failure causes over 80–90% of deaths, but comorbidities include malabsorption, digestive tract cancers, diabetes, renal disease, osteoporosis, cirrhosis, and fertility problems.
Haemophilia	Hamophilia A is the most common and severe form, affecting mainly men (X‐linked). Current life expectancy on average 15 years less than the general population. Associated with spontaneous bleeding (in joints/soft tissues/intestine/brain), leading to acute pain, long‐term disability, or death.
Von Willebrand disease (VWD)	Most common inherited clotting disorder. Affects men and women. Types 1 & 2 generally less severe than haemophilia (lifespan unaffected), type 3 is equivalent to severe haemophilia A.

## Methods

The data for this study were collected as part of a larger sequential mixed methods study exploring the attitudes of people living with genetic conditions towards different types of population screening programmes (pre-conception, PNS, newborn). The analysis presented here focusses on the concept of seriousness among people with SMA, thalassaemia, CF, haemophilia (men/women analysed separately), and FXS, with the addition of people with VWD and FXTAS/FXPOI. Detailed methods can be found in [16, 27, 28]. Ethical approval was granted by the Biomedical and Scientific Research Ethics Committee and the Health Research Authority (17/WM/0231 01/08/17). All participants were informed of the study aims and potential data uses. Interview participants signed a consent form, completion of surveys (anonymously) was deemed a proxy for consent.

### Qualitative data collection and analysis

Forty-five interviews were conducted between March 2017 and January 2019. Thirty-five participants were recruited through condition support groups and 10 through an NHS CF clinic. Interviews were face-to-face (*n* = 14), by telephone (*n* = 29) or email (*n* = 2), as determined by participants’ preferences. Participants were asked about their daily lives with their condition and their attitudes towards screening. Names/identifiers were removed during (verbatim) transcription and replaced with pseudonyms. Open coding (Nvivo 11) identified broad descriptive themes from which subthemes were developed. A conceptual cross-cutting framework was created and organised into a hierarchical framework, utilising an iterative analytic process driven by the data (constant comparison method) with reference to the literature. This was conducted until data saturation was achieved.

### Quantitative data collection using surveys

A core set of questions was developed from the qualitative data (with assistance from condition support groups) with amendments to ensure applicability for each condition. As the survey was originally designed to explore attitudes to hypothetical future screening programmes, questions relating to PNS were not asked of thalassaemia participants due to the pre-existence of a thalassaemia PNS programme in the UK. Surveys (paper/online) were completed between December 2017 and November 2019. Questions were designed to explore attitudes towards screening, but also current health/wellbeing, QoL, suffering, social support, PNS, termination for own condition and the consequences of this (Table 2). Participants responded using five-point likert scales, which were condensed for analysis into positively (agree/strongly agree) or negatively (disagree/strongly disagree) valanced answers and those that were neither (neither/don’t know). Current health and wellbeing responses were condensed into poor/very poor/fair versus good/very good. Statistical differences were determined using Chi squared or Fisher’s Exact (where assumptions of Chi violated) (IBM SPSS Statistics 27). Comparisons across all participants (e.g. relationship between QoL and societal support) used all data combined.

**Table 2:** Summary of attitudes of 469 survey respondents.

		SMA2		SMA3		SMA4		Haemophilia Women		Haemophilia Men		VWD		Cystic Fibrosis		Thalassaemia		FXS		FXTAS/POI	
		(*N* = 28)		(*N* = 31)		(*N* = 23)		(*N* = 29)		(*N* = 151)		(*N* = 77)		(*N* = 21)		(*N* = 61)		(*N* = 18)		(*N* = 30)	
		*N*	%	*N*	%	*N*	%	*N*	%	*N*	%	*N*	%	*N*	%	*N*	%	*N*	%	*N*	%
Current health and wellbeing	Bad/Vb	0	0.0%	2	10.5%	3	16.7%	2	6.9%	24	16.1%	13	17.1%	1	4.8%	3	5.3%	1	5.9%	4	15.4%
	Fair	3	23.1%	7	36.8%	11	61.1%	5	17.2%	53	35.6%	20	26.3%	7	33.3%	21	36.8%	3	17.6%	9	34.6%
	Good/Vg	10	76.9%	10	52.6%	4	22.2%	22	75.9%	72	48.3%	43	56.6%	13	61.9%	33	57.9%	13	76.5%	13	50.0%
It is possible to have a good quality of life	Agree	26	92.9%	23	76.7%	15	65.2%	26	89.7%	131	87.3%	66	86.8%	17	81.0%	48	80.0%	15	83.3%	19	67.9%
	Disagree	0	0.0%	2	6.0%	4	17.0%	0	0.0%	7	4.7%	3	3.9%	0	0.0%	6	10.0%	0	0.0%	5	17.9%
	Dk/neither	2	7.1%	5	16.0%	4	17.4%	3	10.3%	12	8.0%	7	9.2%	4	19.0%	6	10.0%	3	16.7%	4	14.3%
Condition causes people to suffer	Agree	7	25.0%	21	70.0%	16	72.7%	22	75.9%	117	78.5%	50	66.7%	19	90.5%	36	59.0%	25	58.8%	15	53.6%
	Disagree	14	50.0%	4	13.3%	1	4.5%	0	0.0%	11	7.4%	12	16.0%	0	0.0%	14	23.0%	7	17.6%	4	14.3%
	Dk/neither	7	25.0%	5	16.7%	5	22.7%	7	24.1%	21	14.1%	13	17.3%	2	9.5%	11	18.0%	13	23.5%	9	32.1%
People/families well supported by society	Agree	6	21.4%	4	13.3%	1	4.3%	12	41.4%	69	46.0%	20	27.0%	3	14.3%	14	23.0%	7	38.9%	8	28.6%
	Disagree	16	57.1%	20	66.7%	12	52.2%	9	31.0%	41	27.3%	26	35.1%	9	42.9%	31	50.8%	7	38.9%	13	46.4%
	Dk/neither	6	21.4%	6	20.0%	10	43.5%	8	27.6%	40	26.7%	28	37.8%	9	42.9%	16	26.2%	4	22.2%	7	25.0%
I support pre-natal screening	Agree	15	55.6%	25	80.6%	17	77.3%	22	75.9%	84	57.9%	45	60.0%	17	81.0%			15	88.2%	23	76.7%
	Disagree	11	40.7%	4	12.9%	3	13.6%	2	6.9%	20	13.8%	6	8.0%	2	9.5%			1	5.9%	4	13.3%
	Dk/neither	1	3.7%	2	6.5%	2	9.1%	5	17.2%	41	28.3%	24	32.0%	2	9.5%			1	5.9%	3	10.0%
PNS leads to fewer people existing who could have fulfilling lives	Agree	19	67.9%	17	54.8%	14	63.6%	13	44.8%	79	54.9%	32	42.7%	11	52.4%			7	43.8%	16	55.2%
	Disagree	3	10.7%	10	32.3%	4	18.2%	7	24.1%	22	15.3%	15	20.0%	5	23.8%			2	12.5%	4	13.8%
	Dk/neither	6	21.4%	4	12.9%	4	18.2%	9	31.0%	43	29.9%	28	37.3%	5	23.8%			7	43.8%	9	31.0%
Would be loss to have fewer people with condition	Agree	18	64.3%	8	26.7%	6	27.3%	10	34.5%	53	37.1%	23	30.7%	8	38.1%			2	12.5%	11	36.7%
	Disagree	5	17.9%	11	36.7%	6	27.3%	8	27.6%	47	32.9%	20	26.7%	3	14.3%			6	37.5%	9	30.0%
	Dk/neither	5	17.9%	11	36.7%	10	45.5%	11	37.9%	43	30.1%	32	42.7%	10	47.6%			8	50.0%	10	33.3%
PNS enables informed decision making whether to have child	Agree	20	71.4%	25	83.3%	19	86.4%	25	86.2%	103	71.5%	46	62.2%	17	81.0%			10	62.5%	24	80.0%
	Disagree	6	21.4%	0	0.0%	2	9.1%	1	3.4%	18	12.5%	11	14.9%	3	14.3%			1	6.3%	1	3.3%
	Dk/neither	2	7.1%	5	16.7%	1	4.5%	3	10.3%	23	16.0%	17	23.0%	1	4.8%			5	31.3%	5	16.7%
PNS prevents unnecessary suffering	Agree	6	21.4%	16	53.3%	12	54.5%	13	44.8%	52	36.1%	26	34.7%					9	52.9%	12	40.0%
	Disagree	14	50.0%	5	16.7%	6	27.3%	9	31.0%	38	26.4%	19	25.3%					3	17.6%	8	26.7%
	Dk/neither	8	28.6%	9	30.0%	4	18.2%	7	24.1%	54	37.5%	30	40.0%					5	29.4%	10	33.3%
Selective terminaton for condition acceptable	Agree	3	11.1%	10	33.3%	6	27.3%	4	13.8%	17	11.8%	10	13.3%	3	14.3%			2	12.5%	13	46.4%
	Disagree	18	66.7%	10	33.3%	12	54.5%	21	72.4%	94	65.3%	47	62.7%	12	57.1%			5	31.3%	7	25.0%
	Dk/neither	6	22.2%	10	33.3%	4	18.2%	4	13.8%	33	22.9%	18	24.0%	6	28.6%			9	56.3%	8	28.6%

Grey shading signifies question not asked of group.

*PNS* prenatal screeening, *Vb/Vg* very bad/very good, *Dk* don’t know.

### Mixed‐methods data analysis of the concept of seriousness

Quantitative findings from the surveys were cross-referenced with related conceptual themes from the qualitative data during mixed methods analysis to offer context and greater depth of understanding of statistical findings. Representative qualitative excerpts of key findings presented in this paper were selected during this mixed methods analysis.

## Results

There were 469 survey returns and 45 interview participants. Women were over-represented (Table 3), except in SMA4 (survey) and haemophilia (survey and interview) groups. Participants were predominantly of white British ethnicity, except for the thalassaemia group (Asian/Arab, or white European). Survey participants in CF, thalassaemia, SMA2 and FXS groups tended to be younger than the other groups.

**Table 3:** Demographics of 469 survey and 45 interview respondents.

		SMA2			SMA3			SMA4			Haemophilia Women		Haemophilia Men			VWD			Cystic Fibrosis			Thalassaemia			FXS			FXTAS/POI		
		Survey		Interview	Survey		Interview	Survey		Interview	Survey		Survey		Interview	Survey		Interview	Survey		Interview	Survey		Interview	Survey		Interview	Survey		Interview
		*N* (28)	%	*N* (8)	*N* (31)	%	*N* (5)	*N* (23)	%	*N* (2)	*N* (29)	%	*N* (151)	%	*N* (7)	*N* (77)	%	*N* (3)	*N* (21)	%	*N* (10)	*N* (61)	%	*N* (8)	*N* (18)	%	*N* (1)	*N* (30)	%	*N* (1)
Gender	Female	20	71.4	5	17	54.8	4	8	34.8	1	29	100	-		-	61	79.2	3	14	66.7	5	42	68.9	5	12	66.7	1	25	83.3	1
	Male	8	28.6	3	14	45.2	1	15	65.2	1	-		151	100	7	16	20.8	0	7	33.3	5	18	29.5	3	5	27.8	0	5	16.7	0
	NA		0			0			0								0			0			0		1	5.6			0	
Age	Under 35 yrs	11	39.3	4	2	6.5	0	2	8.7	0	4	13.8	23	15.2	1	18	23.4	1	14	66.7	6	16	26.2	1	8	44.4	1	1	3.3	0
	35–55 yrs	14	50.0	3	8	25.8	5	7	30.4	1	14	48.3	47	31.1	3	19	24.7	1	7	33.3	3	39	63.9	7	4	22.2	0	14	46.7	0
	Over 55 yrs	1	3.6	1	14	45.2	0	10	43.5	1	11	37.9	81	53.6	3	40	51.9	1	0	0	1	5	8.2	0	5	27.8	0	15	50.0	1
	NA	2	7.1		7	22.6		4	17.4			0		0			0			0		1	1.6		1	5.6			0	
Ethnicity	Asian/Arab	1	3.6		1	3.2		0	0	1	0	0	2	1.3	0	1	1.3	0	1	4.8	0	33	54.1	5	0	0	0	0	0	0
	Black	0	0	1	0	0		1	4.3	0	0	0	3	2	0	2	2.6	0	1	4.8	0	0	0	0	0	0	0	0	0	0
	White British	26	92.9	7	29	93.5	4	20	87.0	1	29	100.0	137	90.7	7	71	92.2	2	18	85.7	9	0	0	0	16	88.9	1	29	96.7	1
	White Other	1	3.6		1	3.2	1	2	8.7	0	0	0	7	4.6	0	2	2.6	1	1	4.8	1	25	41	3	0	0	0	1	3.3	0
	NA		0			0			0			0	2	1.3		1	1.3			0		3	4.9		2	11.1			0	
Has children	No	18	64.3	7	13	41.9	2	9	39.1	0	3	10.3	59	39.1	2	23	29.9	1	0	0	6	56	91.8	7	13	72.2	1	3	10.0	0
	Yes	10	35.7	1	18	58.1	3	14	60.9	2	26	89.7	92	60.9	5	54	70.1	2	5	23.8	4	4	6.6	1	5	27.8	0	27	90.0	1
	NA		0			0			0			0		0			0		16	76.2		1	1.6			0			0	
Religious faith	No	10	35.7		11	35.5		7	30.4		11	37.9	64	42.4		25	32.5		14	66.7		9	14.8		5	27.8		10	33.3	
	Yes	15	53.6		18	58.1		13	56.5		18	62.1	80	53.0		45	58.4		7	33.3		47	77.0		10	55.6		19	63.3	
	NA	3	10.7		2	6.5		3	13			0	7	4.6		7	9.1			0			0		3	16.7		1	3.3	
Educated to degree level	Yes	17	60.7		5	16.1		8	34.8		10	34.5	65	43.0		38	49.4		13	61.9		28	45.9		2	11.1		15	50.0	
	No	11	39.3		26	83.9		14	60.9		19	65.5	86	57.0		39	50.6		8	38.1		33	54.1		16	88.9		15	50.0	
	NA		0			0		1	4.3			0		0			0			0			0			0			0	

NA = did not answer or ‘prefer not to say’.

### Current health and wellbeing, QoL, and suffering

Participants generally reported good current health, with very few stating they had bad or very bad health/wellbeing (Table 2). Interview participants highlighted how their positive health status could come as a surprise to other people. Samantha (32, CF), who was on the waiting list for a lung transplant, commented, “I think people are surprised when I say I’m doing fine and feel ok most days. Yes, I will have been doing nebulisers all morning and night…but that’s my normal. Perhaps my normal is different to other people’s, but it’s my normal, you know?” This sense of being in good health, despite the perceptions of others, was mirrored across conditions. Participants with SMA2 reported the highest levels of good/very good current/health welfare (76.9%). Mark (25, SMA2), was living in University halls supported by care workers at the time of interview. He was a full-time wheelchair user and used a C-PAP machine to support his breathing. He reflected, “You see, I see myself as really healthy, actually. I don’t often get colds, or have any serious breathing issues or chest infections…I’ve only been hospitalised twice my whole life…[…]… The SMA part is a disability, but it doesn’t impact much on my health actually. It doesn’t make me ill as such, or in much pain…it’s just the way my body is. I don’t think it’s a contradiction to say you’re both healthy and disabled.”

Although not commonly reported, bad or very bad current health/wellbeing was most frequent in participants with VWD (17.1%), SMA4 (16.7%), men with haemophilia (16.1%), and people with FXSTAS/POI (15.4%). Due to their infrequency, these responses were combined with ‘fair’ for pairwise analyses. Participants with SMA4 had the highest levels of bad/fair (77.8%) and lowest levels of good health/wellbeing (22.2%) (Tables 2 & 4), which is striking as SMA4 is clinically the mildest form of SMA with late onset (usually after 35 years). Helen (45) described the dramatic changes in her life and health brought about by SMA4; “I’ll never forget the day I was diagnosed…[…]… I was 37, just got all my kids in school and had just started in a new job …[…]…It’s been downhill from there really, I’m 45 now and I still walk, but…with a frame now. I couldn’t keep up with the job and had to chuck it in which was devastating and that hit us…financially. I’ve just…lost my independence really. Most days I’m so weak I struggle with everything now, it’s a terrible illness….So I would say my health is pretty bad now.” Helen incorporated her SMA into her evaluation of her own health, viewing it as an ‘illness’. In contrast, Mark drew a conceptual distinction between his disability—which had been a comparatively stable presence since infancy—and his health, which he interpreted as being the absence of illnesses, pain and suffering.

**Table 4:** Significant differences in attitudes between survey respondents of different condition groups (Pairwise comparison, signficant differences *p* < 0.05 in bold, trends of *p* ≤ 0.10 also included).

Current health and wellbeing										
	SMA2	SMA3	SMA4	Haem Women	Haem Men	VWD	Cystic Fibrosis	Thalassaemia	FXS	FXTAS/POI
SMA2		.	**0.003**	.	**0.048**	.	.	.	.	0.107
SMA3			0.057	0.095*	.	.	.	.	.	.
SMA4				**0.000**	0.036*	0.009*	**0.013**	**0.008**	**0.001**	0.063
Haem Women					0.007*	0.069*	.	0.101	-	**0.047**
Haem Men						.	.	.	0.028*	.
VWD							.	.	.	.
Cystic Fibrosis								.	.	.
Thalassaemia									.	.
FXS										0.083*
It is possible to have a good quality of life										
	SMA2	SMA3	SMA4	Haem Women	Haem Men	VWD	Cystic Fibrosis	Thalassaemia	FXS	FXTAS/POI
SMA2		.	**0.014**	.	.	.	.	.	.	**0.042**
SMA3			.	.	.	.	.	.	.	.
SMA4				**0.037**	0.017*	**0.028**	.	.	.	.
Haem Women					.	.	.	.	.	**0.042**
Haem Men						.	.	.	.	**0.015**
VWD							.	.	.	**0.035**
Cystic Fibrosis								.	.	.
Thalassaemia									.	.
FXS										.
The condition causes people to suffer										
	SMA2	SMA3	SMA4	Haem Women	Haem Men	VWD	Cystic Fibrosis	Thalassaemia	FXS	FXTAS/POI
SMA2		0.002*	0.001*	0.000*	0.000*	0.000*	0.000*	0.009*	0.046*	0.013*
SMA3			.	.	.	.	.	.	.	.
SMA4				.	.	.	.	.	.	.
Haem Women					.	0.067*	.	0.019*	0.096	0.073
Haem Men						0.087*	.	0.003*	0.101	**0.016**
VWD							0.081*	.	.	.
Cystic Fibrosis							.	**0.013**	**0.037**	**0.017**
Thalassaemia									.	.
FXS										.
People with condition and families well supported by society										
	SMA2	SMA3	SMA4	Haem Women	Haem Men	VWD	Cystic Fibrosis	Thalassaemia	FXS	FXTAS
SMA2		.	.	.	0.006*	.	.	.	.	.
SMA3			.	0.015*	0.000*	0.013*	.	.	0.101	.
SMA4				0.009*	0.001*	**0.06**	.	0.089	**0.027**	0.053
Haem Women					.	.	.	.	.	.
Haem Men						.	0.022*	0.002*	.	0.104
VWD							.	.	.	.
Cystic Fibrosis								.	.	.
Thalassaemia									.	.
FXS										.
I support prenatal screening										
	SMA2	SMA3	SMA4	Haem Women	Haem Men	VWD	Cystic Fibrosis	Thalassaemia	FXS	FXTAS
SMA2		**0.041**	0.088	**0.004**	0.001*	0.000*	**0.027**		**0.017**	**0.048**
SMA3			.	.	0.028*	0.020*	.		.	.
SMA4				.	.	0.095	.		.	.
Haem Women					.	.	.		.	.
Haem Men						.	.		0.052	0.094
VWD							.		0.066	0.061
Cystic Fibrosis									.	.
Thalassaemia										
FXS										.
Pregnancy screen will lead to fewer people existing who could have lived fulfilling lives										
	SMA2	SMA3	SMA4	Haem Women	Haem Men	VWD	Cystic Fibrosis	Thalassaemia	FXS	FXTAS
SMA2		.	.	.	.	0.075	.		.	.
SMA3			.	.	0.034*	0.040*	.		0.059	0.107
SMA4				.	.	.	.		.	.
Haem Women					.	.	.		.	.
Haem Men						.	.		.	.
VWD							.		.	.
Cystic Fibrosis									.	.
Thalassaemia										
FXS										.
Loss to society to have fewer people with condition										
	SMA2	SMA3	SMA4	Haem Women	Haem Men	VWD	Cystic Fibrosis	Thalassaemia	FXS	FXTAS
SMA2		0.016*	0.028*	0.074	0.028*	0.007*	0.071		**0.003**	0.109
SMA3			.	.	.	.	.		.	.
SMA4				.	.	.	.		.	.
Haem Women					.	.	.		.	.
Haem Men						.	.		.	.
VWD							.		.	.
Cystic Fibrosis									.	.
Thalassaemia										
FXS										.
Pregnancy screen enables informed decision about having child with condition										
	SMA2	SMA3	SMA4	Haem Women	Haem Men	VWD	Cystic Fibrosis	Thalassaemia	FXS	FXTAS
SMA2		**0.022**	.	.	.	.	.		.	0.087
SMA3			.	.	.	0.044*	0.061		.	.
SMA4				.	.	0.094	.		0.083	.
Haem Women					.	0.055	.		.	.
Haem Men						.	.		.	.
VWD							.		.	.
Cystic Fibrosis									0.1	.
Thalassaemia										
FXS										.
Pregnancy screen will prevent unnecessary suffering										
	SMA2	SMA3	SMA4	Haem Women	Haem Men	VWD	Cystic Fibrosis	Thalassaemia	FXS	FXTAS
SMA2		0.012*	0.052	.	0.042*	0.057			0.048*	.
SMA3			.	.	.	.			.	.
SMA4				.	.	.			.	.
Haem Women					.	.			.	.
Haem Men						.			.	.
VWD									.	.
Cystic Fibrosis										
Thalassaemia										
FXS										.
Selective termination for condition acceptable										
	SMA2	SMA3	SMA4	Haem Women	Haem Men	VWD	Cystic Fibrosis	Thalassaemia	FXS	FXTAS
SMA2		0.031*	.	.	.	.	.		**0.047**	0.003*
SMA3			.	0.011*	0.002*	0.014*	.		.	.
SMA4				.	.	.	.		0.063	0.102
Haem Women					.	.	.		**0.007**	0.001*
Haem Men						.	.		**0.012**	0.000*
VWD							.		**0.028**	0.000*
Cystic Fibrosis									.	0.030*
Thalassaemia										
FXS										0.061

*Haem* haemophilia.

Pairwise comparisons conducted using Chi squared (*), except where assumptions violated and Fisher’s Exact used.

Nearly all participants agreed that it is possible to have a good QoL with the condition they lived with; however, in a similar pattern to that observed with health, participants with SMA2 were most likely to agree (92.9%) and participants with adult onset conditions least likely to agree (SMA4 65.2%, FXTAS/POI 67.9%). This difference between early and later onset conditions was also evident within the qualitative dataset. James (46, haemophilia) had received regular treatment with blood products from childhood and through this, contracted Hepatitis C at age 10. The hepatitis was successfully treated, but James’s haemophilia led to arthritis, prompting him to resign from his active job. He reflected, “Quality of life … is what you make it really. Yes, I’ve got my issues to deal with, but so does everyone, just in different ways…although some people will tell you you need your health to have a quality of life, I think I’m living proof that you can have it anyway. As long as you have positive relationships, you know, you have things to look forward to, you have things to do, that’s all you really need.” The qualitative data revealed that participants, such as James, who had lived with their condition since birth or childhood—even though his condition had changed over time and had implications for health as well as disability—were more likely to define QoL as a combination of factors external to themselves, such as social relationships, interests and activities, which haemophilia did not preclude. This was in stark contrast to participants with later onset conditions, such as Mary (74), diagnosed with FXTAS in her early 60 s who commented on the onset of her condition, “My quality of life deteriorated considerably. Everything you take for granted in life gets pulled out from under you, like driving, shopping, cooking a meal, even basic things like drinking a hot drink without spilling it all over yourself….you shake constantly and feel tired constantly…[…]…and when you can’t do those things no more—there isn’t much quality left is there?”. For Mary, health, QoL and FXTAS were inextricably bound together. Having once enjoyed life, Mary’s FXTAS was a restrictive, unwelcome and unexpected burden, prompting a re-evaluation of her lifestyle and identity, akin to what Bury has refereed to ‘biographical disruption’ and re-integration [29].

Differences between early and later onset conditions were less pronounced, however, within perceptions of suffering. Almost all of those with CF (90.5%) agreed that CF causes suffering (Table 2), which was significant compared with SMA2 (25.0%), thalassaemia (59.0%), FXS (58.8%) and FXTAS/POI (53.6%) (Table 4). After CF, the greatest proportion of participants reporting suffering were those with haemophilia (men 78.5%, women 75.9%), SMA3 (70.0%) and SMA4 (72.7%). This seeming contradiction, that genetic conditions cause suffering, but that a good QoL can be achieved, was expressed by the majority of participants within the qualitative data. Johara (36, thalassaemia) commented, “Thalassaemia is hard…I am ill a lot, I get tired, and I have restrictions in my life because of it….but that doesn’t mean that you can’t have a good life..[…]….I’d always choose to be here, even with thalassaemia.”

### Societal influences

Across all conditions, fewer than 50% of respondents felt well supported by society Men with haemophilia most frequently (although still only 46%) felt well supported, and respondents with SMA3 (13.3%) and SMA4 (4.3%) least frequently felt supported. Within the qualitative data, links between societal support and lived experience were made explicit, with many participants emphasising that the social consequences of their condition—and its interface with their environmental context—could be as disabling as the physiological effects of a genetic condition, as highlighted by Olivia (37, SMA2), “I don’t much think about having SMA, what I think more about is the access issues. As long as society is accessible, I’m not restricted…But if I can’t get into a building, if I can’t get around…yeah, that’s when I’m disabled.” Indeed, within the survey, of the 27 participants who felt that good QoL was not possible with their condition, 77.8% felt unsupported by society (compared with 35.8% of the 385 people who thought a good QoL was possible, Chi = 23.63 *p* < 0.001), suggesting that social factors play an important role in negative lived experience.

### Attitudes towards PNS and pregnancy termination

There were high levels of support for PNS from people with FXS (88.2%), CF (81.0%), SMA3 (80.6%), SMA4 (77.3%), and FXTAS/POI (76.7%) (Tables 2 & 4). Men with haemophilia and people with VWD showed lower levels of support (57.9% & 60.0%) but were more likely to select don’t know/neither (28.3% & 32.0%) than disagree (13.8% & 8.0%). People with SMA2 were least in favour of PNS (55.6%), and most likely to select disagree (40.7%). Enabling informed-decision making about whether to have an affected child (62–86% across groups) appeared to be a key driver of support for PNS. Isabelle (26, FXS), commented, “I don’t think it’s [PNS and pregnancy termination] a problem as it’s the parents’ choice. I think they have a right to choose not to have a child with fragile X if they don’t want to. It’s important to have full choice and…be fully aware.” Other interview participants highlighted the importance of fully committed parents to successfully raise a child with additional support needs, seeing PNS as a way to offer a ‘way out’ (David, 47, CF) to parents who feel they could not cope. Despite this, there was little support for selective pregnancy termination for their condition (<15% agreement for most conditions). The exceptions to this were FXTAS/POI and SMA3 groups (46.4% and 33.3%, respectively), and also SMA4 (27.3%, not significant). The interview data highlighted the complexity of this issue for people living with genetic conditions. Illona (23, VWD) reflected, “I support it [PNS], but I don’t think I’d ever use it myself. I’m very much pro-choice so I think the parents are entitled to make that choice… But, would I feel comfortable doing it? No…[…]…because I have a genetic condition myself, so I couldn’t abort a baby for having the same as me. It would make me a hypocrite.”

There was a high degree of ambivalence in responses to the statement ‘PNS will lead to fewer people with [the condition] coming into the world who could have lived fulfilling lives’, with 53% agreement overall, 18% disagreeing, and the remainder (29%) selecting neither or ‘don’t know’. Responses to whether ‘it would be a loss to society to have fewer people with the condition’ revealed even greater ambivalence, with significantly fewer people in support than in response to the question regarding fulfilling lives (35%, Chi = 52.74 *p* < 0.0001). Overall, 26% of all participants agreed with both statements, whereas 48% answered neither/don’t know to at least one. Alex (46 years, haemophilia A), endorsed the viewpoint that people with his condition have much to contribute, “People with haemophilia have a lot to offer the world. They come with a unique perspective…. They live with something that’s not quite the norm…and we need people like that…they have insights and understandings that we need more of … not less.”

Participants with SMA2, again, differed from the other groups, being most likely to consider it a loss to society to have fewer people with their condition coming into the world (64.3%) and that screening would prevent people being born who could have lived fulfilling lives (67.9%; 57% agreeing with both statements). Participants with SMA2 (who also perceived the lowest levels of suffering) were also least likely to agree (21.4%) and most likely to disagree (54.5%) with the statement ‘PNS can prevent unnecessary suffering’. Claire (30 years, SMA2), commented, “I don’t think SMA causes suffering… Perhaps the parents suffer because they were thinking their child would be fine.…Certainly I don’t know anyone with type II who would say they’re suffering… I think that’s perhaps just an idea about disability that people put on us …. unnecessarily, you know [laughs] because we’re fine!”

## Discussion

This paper is, to the best of our knowledge, the largest to explore attitudes and experiences relevant to the concept of condition ‘seriousness’ and PNS/selective termination exclusively from the perspectives of 514 adults living with clinically ‘serious’, and widely contrasting, genetic conditions. When set in the broader literature, all of the included conditions (with the exception of haemophilia which wasn’t subject to the algorithm) were classified as ‘severe’ using Lazarin et al’s taxonomy [5, 13], a system designed to classify conditions to simplify reproductive decision-making for prospective parents. In addition, all five are included on the pre-conception population screening panel as part of the McKenzie’s Mission pilot (Australia), based on their seriousness, treatment availability and whether an ‘average’ couple could reasonably be expected to want to avoid having a child with that condition [3]. One key finding of this study is, however, that whilst experiencing a clinically serious condition, the vast majority of participants reported both good health and the possibility of having a good QoL. Assuming that health/wellbeing/QoL are core dimensions of a condition’s seriousness, these findings indicate notable differences between the lived experience of affected adults and the way genetic conditions are defined and understood in the context of reproductive screening programmes. This divergence illustrates the importance of including the views and lived experiences of those directly affected by genetic conditions in ethical debates surrounding the scope, and practical implementation of screening programmes. More specifically, debates around which conditions are considered ‘serious’ enough to qualify them as candidates for screening, as well as those around the adequacy and quality of informed consent for screening and any subsequent (prenatal) diagnosis. This is particularly important in relation to rare genetic conditions where public awareness, and knowledge of their lived realities, is typically low.

It has been suggested that people with genetic conditions might use different frames of reference for assessing their own health/wellbeing vis-à-vis those without genetic conditions [23, 24], and there was evidence within the dataset of this e.g. Samantha, CF. Indeed, physical and psychological adaptation to the condition, including its incorporation within personal identity (e.g. Mark, SMA2) was found to play a significant role in the way that health and QoL were interpreted. Participants who did not see their condition as part of themselves were more likely to view it as an illness, separate to their sense of self, that impacted their health and QoL (Mary, FXTAS).

Those with later onset conditions were more likely than those with early onset to report poorer health and consider a good QoL impossible. Previous research suggests that age of onset has a critical role to play in positive adaptation to a condition, and the likelihood of its incorporation into personal identity [30]. Indeed, those with later onset conditions were more likely to experience ‘biographical disruption’ [29] (Helen, SMA4), and associated forms of identity re-formulation around a new health state. This contrast was most clearly demonstrated in relation to SMA, where participants with milder, later onset types (SMA3/4), consistently reported poorer health/wellbeing than those with SMA2, suggesting a lack of correlation between clinical definitions of condition seriousness and lived realities for these adults. Late onset conditions have traditionally been excluded from reproductive screening programmes [26], often being interpreted as less serious (by virtue of the number of condition-free years) [31], yet, our data suggest that those with later onset conditions experience unique challenges in adjusting to their condition, irrespective of clinical seriousness.

The majority of participants perceived poor societal support, which was particularly evident in the minority who did not consider a good QoL to be possible. The influence of social and environmental factors on disease seriousness has been vastly under-explored in the context of reproductive genetics. As Kleiderman et al. [9] outline, the concept of ‘serious disease’ cannot be understood outside of social/environmental contexts—including societal/cultural influences on the interpretation of serious disease, health, and disability, which vary by time and place. However, the social and cultural context in which the condition will be experienced is not accounted for within taxonomies of condition seriousness.

The complexity of the views of people with genetic conditions, their largely positive lived experiences and supportive attitudes towards PNS, but also their resistance to the associated practice of selective termination, all emerged from this study. Participants highlighted that suffering and high quality of life can co-exist without contradiction. Their widespread support for PNS, when interpreted on its own, masks the deeply ambivalent views they held towards reproductive genetic technologies, and the value they still assign to future lives with their condition [18]. When designing taxonomies capable of classifying condition seriousness, future work may usefully take into account this complexity of lived experience, particularly in the context of the emergence of new therapies (e.g. nusinersen for spinal muscular atrophy) that may modify the nature of those experiences for future generations. Mixed methodologies, capable of capturing not only lived experience, but also the way that participants value different aspects of that experience, and balance the relative harms and benefits of screening (e.g. discrete choice experiments, Q methodologies) may prove to be particularly useful in this regard given the multifaceted nature of this topic. Indeed, the age of onset of the condition, its intersection with perceptions of health, disability and identity, and the social and environmental context in which it is experienced, were all found by this study to be fundamentally important to the way that genetic conditions were lived, yet have all been under-explored in debates around condition seriousness. These factors were found to override clinical markers of disease seriousness in their influence on lived experience, suggesting a need for a much wider range of factors and perspectives to be taken into account when determining what constitutes a serious condition. This is particularly important in an age of expanded genomic screening, wherein the conceptual apparatus used to define condition seriousness determines not only access to genomic screening programmes, but also the range of reproductive options available once a condition is found; ultimately influencing who does, and who does not, come into the world.

## Limitations

The study questions included in this analysis were designed to assess attitudes towards hypothetical screening scenarios rather than perceptions of seriousness per se. For this reason, not all of the survey questions were asked of every group (e.g. participants with thalassaemia were not asked about prenatal screening). These data were therefore missing. The self-selecting nature of the sample introduces limitations, in that those who participated may have been in better health, and have more positive perspectives than those who opted not to. Despite this, participants represented a broad range of experiences/clinical severities within/across conditions and the included questions were all highly relevant to an analysis of seriousness. Adults with FXS were, however, underrepresented in the final sample. This was in spite of efforts to remove barriers to interview participation to allow for learning differences (e.g. adapted interview schedule/alternative interview methods, supportive adult present). As some of the reported data date back to 2017, this could also be considered a limitation of the study, given the pace of change, particularly in the field of expanded carrier screening in the international arena. However, as this study offers insights into the lived experiences of adults with a broad range of genetic conditions, their perceptions of their condition’s ‘seriousness’, and ultimately its prevention, the findings retain a high degree of relevance to debates underpinning the inclusion of conditions on such carrier screening panels (e.g. [7]), which are set to continue, even as such programmes begin to be implemented and/or expanded.

## Data Availability

The datasets generated and analysed during the current study are not yet publicly available but will be deposited at the end of the study. However, they are available from the corresponding author on reasonable request.
